# Lumbar Epidural: Anatomical and Clinical Study in Dogs Submitted to Ovariohysterectomy

**DOI:** 10.3389/fvets.2020.527812

**Published:** 2020-11-03

**Authors:** Daniela Santilli Cima, Leonardo de Freitas Guimarães Arcoverde Credie, Fábio Futema, Stelio Pacca Loureiro Luna

**Affiliations:** ^1^Department of Anesthesiology, Medical School, São Paulo State University (Unesp), Botucatu, Brazil; ^2^School of Veterinary Medicine, São Judas Tadeu University, Santos, Brazil; ^3^School of Veterinary Medicine, São Judas Tadeu University, São Paulo, Brazil; ^4^Department of Veterinary Surgery and Animal Reproduction, School of Veterinary Medicine and Animal Science, São Paulo State University (Unesp), Botucatu, Brazil

**Keywords:** analgesia, anesthesia, dog, local anesthetic, locoregional anesthesia, lumbar epidural anesthesia, neuroaxis, pain

## Abstract

Epidural anesthesia minimizes perioperative pain in dogs. It is considered that epidural solution dispersion in cadavers is similar to alive dogs. The objective of the anatomical study was to compare the dispersion of 0.2 mL/kg 0.25% bupivacaine and iohexol via lumbar epidural (L1–L2) under fluoroscopic guidance in 10 thawed cadavers (GC) and 13 female dogs (G0.25) (5–15 kg; body score 4/5). The objective of the clinical study was to evaluate postoperative analgesic consumption and sedation for 6 h after extubation of dogs submitted to ovariohysterectomy when using 0.25% (G0.25; *n* = 10) bupivacaine with the intraoperative use of fentanyl (GF; *n* = 10). Parametric data were compared by the *t*-test and non-parametric data by the Mann Whitney test. Pain and sedation scores were evaluated over time by the Friedman test, followed by the Dunn test. Alive dogs presented greater epidural dispersion (17 ± 3 vertebrae) than thawed cadavers (11 ± 4 vertebrae; *p* = 0.002). All dogs treated with fentanyl and only one dog treated with 0.25% epidural bupivacaine presented pain scores above the cut-off point of the Glasgow Composite Measure Pain Scale Short-Form (GCMPS-SF) and required postoperative rescue analgesia up to 6 h after extubation. The sedation score was higher at all postoperative moments compared to preoperative moments in the G0.25 and GF, except for evaluations performed at 5 and 6 h after extubation in the GF. Greater sedation was observed immediately after extubation in the GF compared to the G0.25, and there was greater sedation in the G0.25 compared to the GF from 3 to 6 h after extubation. The conclusion of the anatomical study was that L1–L2 epidural bupivacaine dispersion is lower in canine thawed cadavers than in alive dogs. Conclusion of the clinical study was that lumbar epidural anesthesia improved postoperative analgesia and produced longer postoperative sedation when compared to fentanyl.

## Introduction

Lumbosacral epidural anesthesia is indicated in surgeries caudal to the diaphragm (1, 2) as it promotes perioperative analgesia, reduces the metabolic, and endocrine response to surgical stress (3), expedites anesthetic recovery, and reduces the requirement for general anesthetics and opioids and, thus, their adverse effects (1, 2, 4).

Epidural anesthesia is usually performed in the lumbosacral region in dogs. The volume of local anesthetic ranges from 0.1 to 0.2 mL/kg (1, 5); the blockade area is limited to the region caudal to the umbilical scar since it provides only lumbosacral plexus anesthesia (L3 to S1). Other *in vivo* epidural puncture sites have been proposed, such as T11 to L1, L6 to S1 (6, 7), and sacrococcygeal (7).

Occasionally it is necessary to anesthetize the cranial abdominal region, innervated by sensitive nerves emerging from thoracic vertebra 8 (T8) to lumbar 3 (L3) (8). Neuraxial anesthesia of the cranial and middle abdomen is still a challenge. Although the technique of epidural space puncture between L1 and L2 has already been described (9), there are no known publications on its clinical application for abdominal surgeries. Other options include transverse abdominis plane block, which provides skin and abdominal muscle anesthesia for mastectomy (10), however, its effectiveness in intra-abdominal surgeries is unknown.

In humans, numerous factors influence the dispersion of drugs following epidural administration, modifying the pattern of sensory blockade. Among those related to the technique itself are patient position, puncture site, solution volume, local anesthetic concentration, and injection rate. The patient-related factors are compartmentalization by meningo-vertebral ligaments, epidural space pressure, which alters through abdominal and thoracic pressure transmitted by intervertebral foramina, the amount of adipose tissue and vessels in the epidural space, and anatomical variations (1, 11–15).

*Post mortem* anatomical studies in cats and dogs have reported cranial progression of methylene blue solution injected into the epidural space after dissection or by means of epidurography at different volumes (11, 14). However, in cadavers, factors that influence epidural dispersion of drugs *in vivo* are not present, among them the lymphatic and circulatory systems, responsible for the continuous movement of solutions administered in the epidural space.

It is generally inferred that the *post mortem* dispersion of dyes in the epidural space follows the same pattern as in alive animals and therefore results can be applied clinically. Based on this affirmative hypothesis, the objective of the anatomical study was to compare, through epidurography, the dispersion of iohexol-associated 0.25% bupivacaine administered via lumbar epidural (L1–L2) in thawed canine cadavers and alive dogs. The objective of the clinical study was to evaluate postoperative analgesic consumption and sedation of female dogs submitted to ovariohysterectomy when using 0.25% bupivacaine in relation to the intraoperative use of fentanyl.

## Materials and Methods

The study was approved by the Animal Ethics Committee of the School of Veterinary Medicine and Animal Science, University of São Paulo State (Unesp), under protocol 0162/2017. Experimental procedures were performed with dogs referred to the Vet Quality Veterinary Hospital, and all owners agreed to the study and signed the informed consent form.

### Anatomical Study (Solution Dispersion)

#### Experimental design

The dispersion pattern of 0.2 mL/kg of a solution combining 0.1 mL/kg 0.5% bupivacaine associated with 0.1 mL/kg iohexol (300 mg/mL), with a final concentration of 0.25% bupivacaine, administered through an L1–L2 epidural approach under fluoroscopy guidance, was evaluated in 13 alive dogs (G0.25) and 10 thawed canine cadavers (GC). The volume and concentration of bupivacaine used were established from a pilot study.

#### Animals

Ten thawed canine cadavers were used, whose deaths occurred due to natural causes or euthanasia in cases where the clinical framework of the animal was incompatible with life, with the consent of the owner, and 13 female dogs were selected for elective ovariohysterectomy and also included in the clinical study described later.

As inclusion criteria, the dogs were required to weigh 5–15 kg and have a body score of 4–5 (16). The alive dogs were required to be from 1 to 5 years of age.

One week before the surgical procedure, blood count, and a serum biochemistry profile were performed (urea, creatinine, alanine aminotransferase, and alkaline phosphatase) as well as a complete clinical examination to ensure the health of the dogs. Dogs with any clinical or laboratory abnormalities, those with skin lesions at the puncture site, and aggressive dogs were excluded. Water and food fasting were instituted for 6 and 8 h, respectively.

The cadavers were thawed at room temperature. Thawing was guaranteed through a similar body surface temperature to the environment (around 24°C) and the possibility of limb movement and positioning of the cadaver for the epidural injection, just as in an alive animal.

#### Lumbar Epidural Anesthesia

Except for antisepsis and anesthesia, all procedures were the same for cadavers and alive dogs. After anesthetic induction, trichotomy, and antisepsis of the thoracic region, the dogs were positioned in sternal recumbency with the pelvic limbs cranially extended. To locate the puncture space, the 13th rib was palpated to identify the last thoracic vertebra and the L1 and L2 spinous processes. Additionally, the lumbosacral space was palpated to identify the seventh lumbar vertebra (L7) and the spinous processes of each lumbar vertebra were palpated up to the space between L1 and L2.

Prior to epidural puncture, fluoroscopic imaging was performed, with contrast (kilovolt) and density (milliamperage) configured to ensure good image visualization. Radiographs were taken of the entire spine in the latero-lateral and dorsoventral views, with the control images obtained before administration of the solution.

The same anesthesiologist performed all epidural injections. A 22G × 50 mm Tuohy needle was used for epidural puncture, introduced from the right side by the paramedian oblique approach, with an angle of ~15° to the spinous process (midline), with the tip of the needle directed to the midline and in a cranial direction, and 45° to the skin. To verify the location of the needle tip in the midline and between L1 and L2, the fluoroscope was positioned vertically at an angle of 90° in relation to the operating table, obtaining dorsoventral images. Subsequently, the fluoroscope was rotated horizontally, at an angle of 0° in relation to the operating table, to monitor the depth of the needle during its introduction in a latero-lateral view. With the bevel directed cranially, the needle passed the skin, subcutaneous tissue, epaxial muscles, and intertransverse ligament. After the crackling sensation of the needle passing the intertransverse ligament, the stylet was removed, and a hanging drop with saline solution was placed. The needle was introduced until the second crackling sensation (yellow ligament), when the hanging drop aspiration and confirmation of needle placement in the epidural space could occur. For further confirmation of the lack of resistance, an intravenous set connected to a saline solution bag coupled to the Tuohy needle was used to verify the infusion of three to four drops of solution. The needle puncture and path of the needle was further confirmed in real time by fluoroscopy, used in two different planes in order to check the dorsoventral position of the needle. The syringe was attached to the needle and, after aspiration of the syringe to ensure no liquor or blood reflux, the solution was injected for 60 s. Fluoroscopic imaging confirmed the contrast path and correct positioning of the needle bevel in the epidural space. Immediately and 5 min after epidural injection, radiographs of the entire spine were performed with latero-lateral and dorsoventral views.

To establish contrast dispersion, images taken before and 5 min after administration of the solution were compared and analyzed by counting the vertebrae until no contrast was observed in the epidural space. In radiographs taken in the latero-lateral view, only the vertebrae with more than 50% of their vertebral body affected by contrast in the ventral portion were calculated. Control images obtained before epidural anesthesia were used to compare and confirm the presence of contrast to avoid technique overlaps or artifacts. Thus, the total number of vertebrae reached by the solution, as well as the cranial and caudal progression, calculated from L1, were established to compare the alive dogs and thawed canine cadaver results.

The dogs were positioned in dorsal recumbency to perform the surgery at the end of the puncture and the cadavers were sent to the hospital disposal service.

### Clinical Study

#### Dogs

Twenty-three bitches, classified as ASA I (American Society of Anesthesiologists), selected for elective ovariohysterectomy, were used according to the same criteria described above for the anatomic group.

Of these bitches, 13 were also part of the anatomical study that evaluated dispersion through epidurography, and after surgery, their postoperative pain and sedation scores were assessed for comparison with the positive control group.

#### Experimental Groups

A group of 13 dogs (G0.25) was submitted to epidural anesthesia under fluoroscopic guidance as described before. This group was composed of the same bitches of the anatomical study. Other 10 dogs included in the positive control group (GF) received 2 μg/kg intravenous (IV) fentanyl 2 min before skin incision [5 μg/mL diluted with lactated Ringer's solution (17, 18)].

#### Anesthesia and Intraoperative Monitoring

On the day of surgery, after the physical examination, the dogs were treated with 0.05 mg/kg acepromazine intramuscularly (IM) and 0.2 mg/kg of meloxicam IM, followed 30 min later by induction of anesthesia with propofol IV and intraoperative IV infusion with 2 mL/kg/h of lactated Ringer's solution. After orotracheal intubation, anesthesia was maintained with isoflurane in a mixture of 80% O_2_ and 20% compressed air using a calibrated vaporizer and anesthetic circuit compatible with the weight of the dog.

Intraoperative heart and respiratory rates, capnography, and peripheral oxyhemoglobin saturation were monitored. Invasive blood pressure was measured by a catheter inserted into the pedal artery in dogs submitted to epidural anesthesia. Arterial blood pressure was measured by a Doppler in dogs from the GF.

*Boluses* of fentanyl (1 μg/kg IV) were administered every 60 s when the heart and/or respiratory rates, and/or SABP were 20% above pre-incisional values until the parameters were reduced to pre-incisional values. If the autonomic response was attributed to the superficialization of anesthesia, the concentration of inhalant anesthetic was increased.

#### Postoperative Analgesia and Sedation

In the postoperative period, the female dogs were individually maintained in 160 cm long, 61 cm wide, and 56 cm high stainless steel cages, internally lined with a hygienic carpet, located in the postoperative recovery room (2.6 × 2 m^2^).

Postoperative pain was evaluated by the GCMPS-SF (19) and sedation by a simplified sedation scale (20) before sedation, immediately after extubation, and 0.5, 1, 2, 3, 4, 5, and 6 h after extubation.

After surgery, the bitches with a GCMPS-SF score ≥6/24 and a sedation score <4 received rescue analgesia with 0.5 mg/kg morphine IM. Those that presented a sedation score ≥4/12, without the ability to stand, were rescued when the pain score was ≥5/20. After 30 min, if the scores had not reduced to below the rescue cut-off score, 0.5 mg/kg morphine plus 25 mg/kg dipyrone IM were administered. In the case of a third rescue, 1 mg/kg of ketamine was administered IM. If the pain scores did not reduce, 0.5 mg/kg morphine was administered IM.

The dogs were discharged with a prescription of 25 mg/kg of dipyrone and 5 mg/kg of tramadol BID orally for 5 days.

#### Delayed Postoperative Evaluation

In the group which received the epidural, the owners were contacted by telephone 1, 5, and 10 days after the surgery to evaluate possible complications related to the technique, like sensitivity in the puncture region, lameness, muscle weakness, or difficulty in performing routine activities, such as urinating and defecating.

### Statistical Analysis

Considering a minimal difference of 30% of the total number of vertebrae achieved by the contrast administered into the L1–L2 epidural space in the first five cadavers vs. the first five alive dogs submitted to epidural anesthesia with 0.25% bupivacaine, the minimal number of dogs was 10 for an 80% power of test and α coefficient of 0.05 (http://biomath.info/power/).

The appropriate statistical test was established after analyzing the normality of the data through the Shapiro Wilk test. Normally distributed data were compared between the two groups by the unpaired *t*-test for contrast dispersion. For weight, age, body score, and doses of drugs used in the postoperative period, the data were compared by ANOVA, followed by the Bonferroni test. Comparisons of pain and sedation scores over time within each group were performed by the Friedman test with Dunn's post-test. To compare each moment between the groups, the Mann–Whitney test was performed, as well as for the number of postoperative rescues. The significance level adopted was 5%. Statistical analysis was performed by the Sigma Plot 12.0 software.

## Results

**Figure 1:**
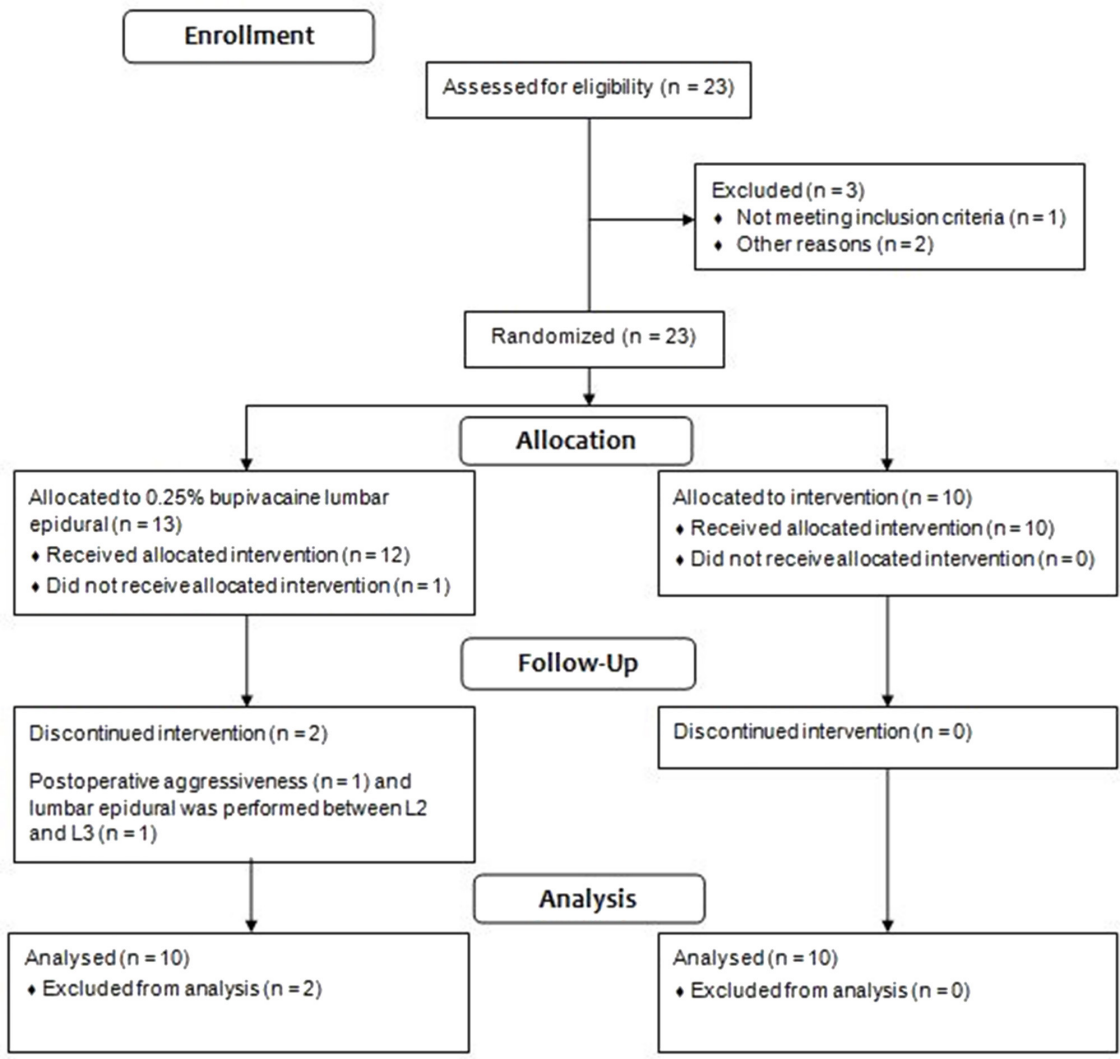
CONSORT flow chart of clinical group allocation and exclusions.

Three of the 13 dogs in the G0.25 were excluded from the study. One bitch was excluded due to presentation of postoperative aggressiveness; one bitch presented high pain scores in the preoperative period due to a reluctance to walk, stiffness, and kyphosis, behaviors attributed to fear; and in one dog the lumbar epidural was performed between L2 and L3, due to a technical difficulty in accessing the space between L1 and L2. Figure 1 contains the flow chart of group allocation and dogs excluded from the study.

The demographic data of dogs from G0.25, GF and GC are shown in Table 1. Supplementary Material contains full data of the study.

**Table 1:** Sex, breeds and mean, standard deviation and *p*-value of age in years, weight in kilograms and body score of dogs undergoing epidural anesthesia with 0.25% bupivacaine (G0.25), or fentanyl bolus (GF) and the cadavers (GC).

	**G0.25**	**GF**	**GC**	***p*-value**
**Age**	2.1 ± 1.15	2.3 ± 1.76	12.2 ± 1.75	<0.0001
**Weight**	8.89 ± 2.99	8.83 ± 3.09	8.84 ± 3.17	0.99
**Body score**	4.5 ± 0.52	4.8 ± 0.42	4.5 ± 0.52	0.30
**Breed**	7 mixed-breed 1 Poodle 1 Lhasa Apso 1 Shih Tzu	7 mixed-breed 1 Teckel 1 Poodle 1 Shih Tzu	2 mixed-breed 1 Teckel 2 Poodle 1 Schnauzer 2 Lhasa Apso 1 French Buldog 1 Shih Tzu	
**Sex**	10 females	10 females	5 females and 5 males	

### Lumbar Epidural Anesthesia

The total number of vertebrae reached by the contrast in the GC and G0.25 is shown in Table 2. Greater dispersion of the solution was observed in the epidural space in the alive dogs than in the thawed cadavers (*p* = 0.002). There were no differences in the dispersion of the solution between images taken immediately and 5 min after the epidural injection. The cranial progression of the solution in the G0.25 was between T7 and C7, and in GC between T13 and C7. The G0.25 presented greater cranial progression than that of GC (*p* = 0.046) (Table 2). The caudal progression of the solution occurred between L2 and S3 in G0.25 and in GC between L2 and L6. Caudal progression was greater in G0.25 than in GC (*p* = 0.013) (Table 2).

**Table 2:** Median [1st; 3rd interquartile range] of total distribution, cranial, and caudal progression in absolute number of vertebrae, after 0.25% bupivacaine and iohexol administered via lumbar epidural between L1 and L2 under fluoroscopic guidance obtained in the anatomical study performed on dogs submitted to ovariohysterectomy (G0.25; *n* = 10) and canine cadavers (GC; *n* = 10).

**Dogs**	**Total distribution**		**Cranial progression**		**Caudal progression**	
	**G0.25**	**GC**	**G0.25**	**GC**	**G0.25**	**GC**
**1**	12	6	8 (T7)	2 (T13)	4 (L5)	4 (L5)
**2**	18	8	12 (T3)	3 (T12)	6 (L7)	5 (L6)
**3**	18	18	13 (T2)	15 (C7)	5 (L6)	3 (L4)
**4**	20	13	15 (C7)	11 (T4)	5 (L6)	2 (L3)
**5**	20	17	15 (C7)	15 (C7)	5 (L6)	2 (L3)
**6**	17	11	13 (T2)	7 (T8)	4 (L5)	4 (L5)
**7**	23	9	14 (T1)	6 (T9)	9 (S3)	3 (L4)
**8**	16	12	15 (T7)	11 (T4)	1 (L2)	1 (L2)
**9**	16	6	11 (T4)	5 (T10)	5 (L6)	1 (L2)
**10**	14	16	10 (T5)	14 (T1)	4 (L5)	2 (L3)
**Median [ ]**	**17 [15; 20]**	**11 [7; 16]**	**13 [11; 15]**	**9 [4; 14]**	**5 [4; 5]**	**2 [2; 4]**

#### Hanging Drop Technique and Lack of Resistance by Drip Infusion

In the G0.25 and GC, the hanging drop technique was positive in 70 and 30% of the dogs, respectively. The lack of resistance by drip infusion was positive in 100% of the alive dogs and in 90% of the cadavers.

### Postoperative Evaluations

**Figure 2:**
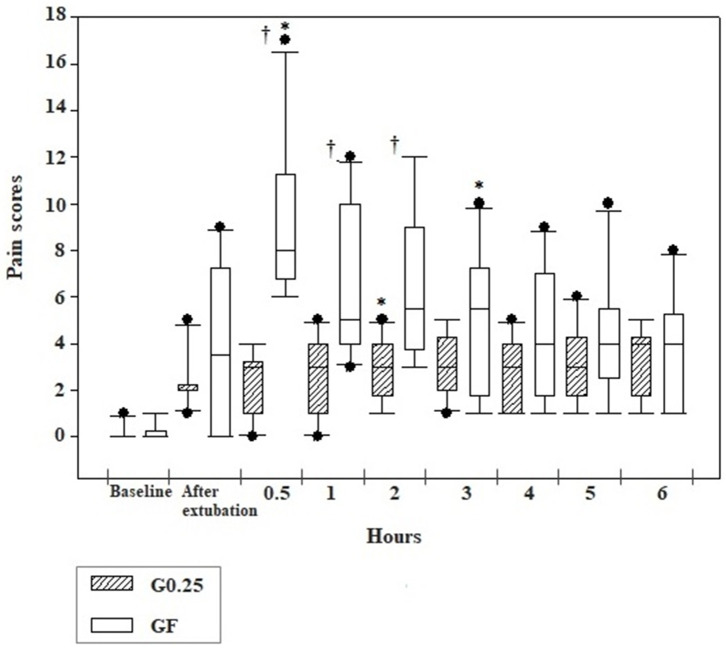
Box plot graphs of scores obtained using the simplified form of the Glasgow University Composite Pain Scale Short-Form in the pre and postoperative periods before sedation (baseline), immediately and 0.5, 1, 2, 3, 4, 5, and 6 h after extubation of dogs submitted to epidural anesthesia with 0.25% bupivacaine (G0.25; *n* = 10) or fentanyl bolus (GF; *n* = 10). The box represents the range corresponding to 50% of the values obtained from the pain score; the line within the box represents the median of pain scores; the line below the median represents the 1st interquartile range and above the median the 3rd interquartile range; the continuous line running through the box comprises data smaller and larger than the 1st and 3rd interquartile ranges, respectively; and the black dots correspond to the atypical values of the pain scores of the samples. *Significant difference from baseline in each group; ^†^ Difference between groups at each time.

Pain scores of the G0.25 increased 2 h after extubation compared to preoperative values, however, they were lower than the cut-off point for analgesic rescue. In the GF, the bitches presented higher pain scores at 0.5 and 3 h after extubation compared to basal values. Dogs treated with fentanyl presented higher pain scores than those receiving 0.25% bupivacaine at 0.5 (*p* <0.0001), 1 (*p* = 0.004), and 2 (*p* = 0.012) hours after extubation (Figure 2).

Postoperative rescue analgesia was performed in all dogs of the GF and only one dog, once, of the G0.25 5 h after extubation (*p* = 0.0002) (Table 3). In the GF, eight dogs received a second rescue analgesia, seven required a third rescue analgesia, and three required a forth rescue analgesia. Morphine consumption was higher in the GF than in the G0.25 (*p* **<** 0.002).

**Table 3:** Median [1st; 3rd interquartile range] of rescue analgesics, and doses of morphine, ketamine, and dipyrone administered postoperatively, in dogs submitted to epidural anesthesia with 0.25% bupivacaine (G0.25; *n* = 10) or fentanyl bolus (GF; *n* = 10).

**Dogs**	**Number of rescue analgesics**		**Morphine (mg/kg)**		**Ketamine (mg/kg)**		**Dipyrone (mg/kg)**	
	**G0.25**	**GF**	**G0.25**	**GF**	**G0.25**	**GF**	**G0.25**	**GF**
**1**	1	2	0.5	1.0	0	0	0	25
**2**	0	1	0	0.5	0	0	0	0
**3**	0	4	0	1.5	0	0.5	0	25
**4**	0	3	0	1.0	0	1	0	25
**5**	0	3	0	1.0	0	1	0	25
**6**	0	4	0	1.5	0	1	0	25
**7**	0	3	0	1.0	0	1	0	25
**8**	0	4	0	1.5	0	1	0	25
**9**	0	1	0	0.5	0	0	0	0
**10**	0	3	0	1.0	0	1	0	25
**Median [ ]**	**0 [0; 0]**	**3 [2; 4]**	**0 [0; 0]**	**1 [1; 1.5]**	**0 [0; 0]**	**0 [0; 1]**	**0 [0; 0]**	**25 [19; 25]**
***p*****-value**	**0.0002**		**<0.001**		**<0.002**		**<0.001**	

**Figure 3:**
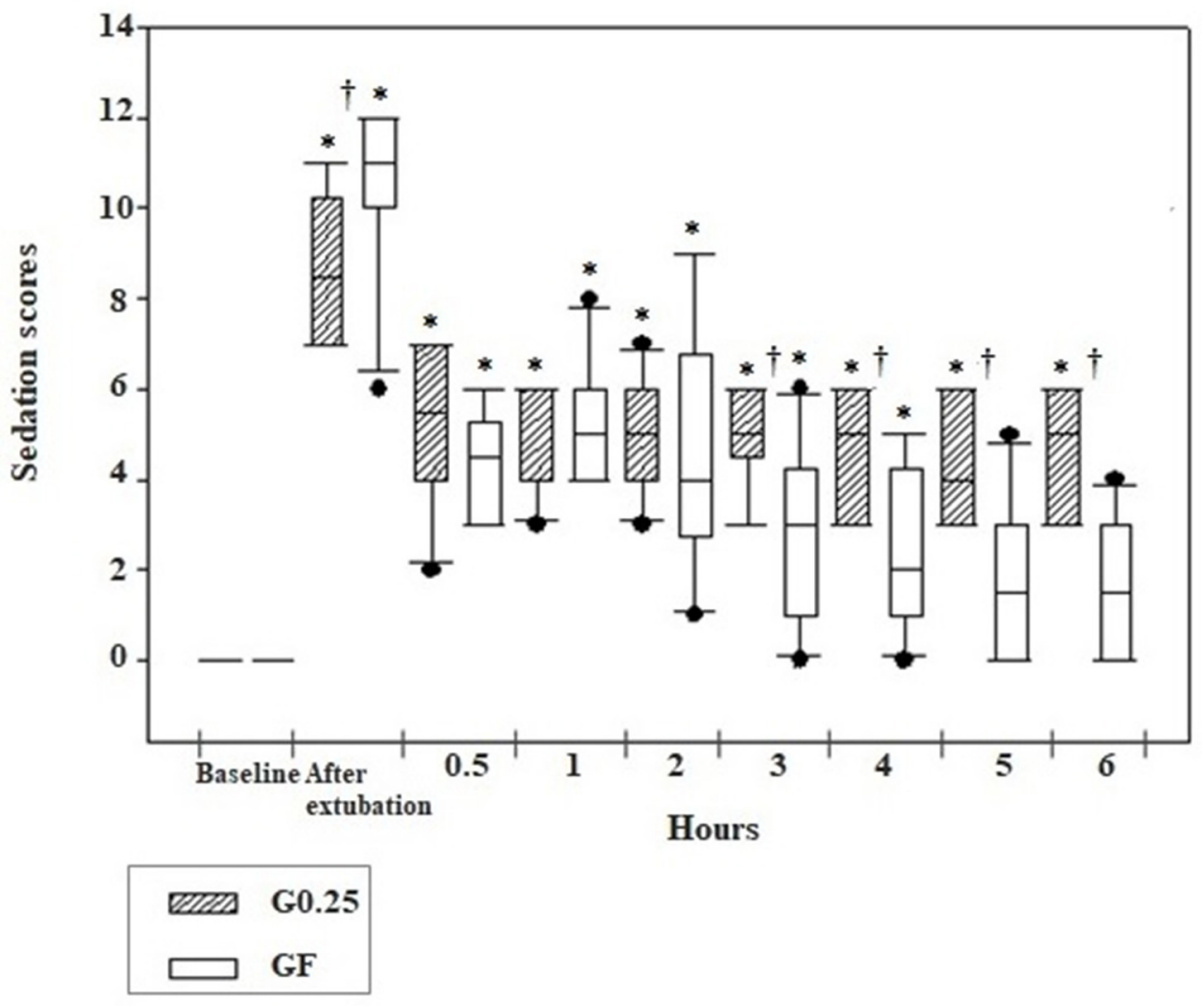
Box plot graphs of scores obtained using the simplified sedation scale in the pre and postoperative period before sedation (baseline), immediately and 0.5, 1, 2, 3, 4, 5, and 6 h after extubation of the dogs submitted to epidural anesthesia with 0.25% bupivacaine (G0.25; *n* = 10) or fentanyl bolus (GF; *n* = 10). The box represents the range corresponding to 50% of the values obtained from the sedation score; the line within the box represents the median of sedation scores; the line below the median represents the 1st interquartile range and above the median the 3rd interquartile range; the continuous line running through the box comprises data smaller and larger than the 1st and 3rd interquartile ranges, respectively; and the black dots correspond to the atypical values of the sedation scores of the samples. *Significant difference from baseline in each group; ^†^ Difference between groups at each time.

The sedation score was higher at all postoperative moments compared to preoperative moments in the G0.25 and GF, except for evaluations performed at 5 and 6 h after extubation in the GF. Greater sedation was observed immediately after extubation in the GF compared to the G0.25 (*p* = 0.02) and there was greater sedation in the G0.25 compared to the GF at 3 (*p* = 0.017), 4 (*p* = 0.012), 5 (*p* = 0.003), and 6 (*p* = 0.001) hours after extubation (Figure 3).

No owners reported discomfort or postoperative complications.

## Discussion

The hypothesis of the study was rejected since the cranial and caudal progression after epidural injection of iohexol-associated bupivacaine administered in L1 and L2 was greater in alive dogs than in thawed canine cadavers. Therefore, at least for lumbar epidural injection, care should be taken when extrapolating the results of studies on cadavers to alive dogs, as the progression of administered solutions may be underestimated. As expected, epidural anesthesia reduced postoperative rescue analgesia of female dogs submitted to ovariohysterectomy.

Factors that influence the dispersion of drugs administered into the epidural space, such as age, pregnancy, body score, puncture site, patient position, needle bevel direction, injection speed, and catheter use or not (12, 13) were controlled in this study, except the age of the cadavers. The older the patient, the smaller the amount of epidural adipose tissue and, consequently, the greater the spread of the local anesthetic (21). Older human patients present higher cranial progression of epidural anesthetics and, therefore, elderly patients require a lower drug volume (13). In the current study, the results were opposite, since the progression in older thawed cadavers was smaller than in younger alive dogs. This suggests that other factors differentiate cadavers from alive dogs and that studies on the dispersion pattern of solutions administered in the thawed cadaver epidural space may not be reliable for clinical use.

The epidural injection volume of 0.2 mL/kg was based on previous publications evaluating the dispersion of contrasts administered via thoracic epidural (6, 14). The administration of iohexol in alive dogs through a catheter inserted between the T11 and L1 vertebrae, with an average tip location at T8, produced a dispersion of 18.5 vertebrae (6), similar to that observed in the alive dogs in the current study and superior to that observed in the cadavers. A possible explanation for this difference is that the absence of epidural pressure generated by the lymphatic and circulatory systems and abdominal and thoracic cavity pressures in cadavers apparently reduces the progression of substances administered by this route (13, 14). Another possible factor might be the possibility of dural sac collapse after freezing, increasing the width of the epidural space, and reducing solution dispersion in defrosted anatomical models.

The characteristics of the injected solution is another factor that could interfere with its progression in the epidural space, however, at least in humans, the viscosity of the substance does not affect solution progression (22). Thus, in theory, the addition of a dense contrast to the solution did not interfere with the epidural progression, therefore contrast progression in epidurography should be the same as local anesthetic progression, to predict blocking effectiveness (23).

Fluoroscopic imaging is the gold standard to confirm the correct positioning of the needle bevel in the epidural space (24), however, as it is expensive and emits high radiation, it is not used in the clinical routine (24, 25). The hanging drop technique and lack of resistance by drip infusion are low-cost option and easy to apply. However, this study corroborated previous research suggesting that the hanging drop technique only confirms epidural puncture in 70–80% of dogs in sternal recumbency and is unsatisfactory in dogs positioned in lateral recumbency (18%) (24–26), and in cadavers, as in the current study (30%). In cattle, lumbar epidural space pressure is negative in standing animals and positive in animals in lateral or dorsal recumbency, a fact attributed to alterations in intra-abdominal pressure due to gravity, which affect the organs of the abdominal cavity and the hydrostatic pressure of the blood vessels, according to the decubitus (27). The *post mortem* pressure changes in the epidural space reduced the effectiveness of the hanging drop technique and the progression of the solution. The lack of resistance, observed by the drip infusion in the intravenous set, on the other hand, showed a sensitivity of 90% in cadavers and 100% in alive dogs in this study.

The analgesic efficacy of this epidural technique in ovariohysterectomy was confirmed since there was better control of postoperative pain in bitches under epidural anesthesia in relation to the use of fentanyl and the results of epidural diffusion of the contrast-containing solution can be extrapolated in relation to the administration of local anesthetic only.

Bitches submitted to the lumbar epidural anesthesia had lower pain scores and need for analgesic rescue compared to control group. Epidural anesthesia reduces postoperative analgesic consumption (1, 28, 29). In the present study, no rescue analgesia was required for 6 h, except for one dog rescued 5 h after surgery, when the effect of the 0.25% bupivacaine had probably finished.

In the current study, epidural anesthesia produced longer postoperative sedation in dogs than the fentanyl treatment, possibly due to descending excitatory modulation by inhibition of the spinal afferent input (30–32). Sedation did not prevent dogs from walking or interacting with the evaluator.

This article followed the recommendations of ARRIVE guidelines (33) except for the following caveats: there was no randomization because the cadavers were selected according to the inclusion criteria and according to the occurrence of death. The alive dogs were allocated to the epidural group whenever the fluoroscope was available for use, otherwise, they were treated with fentanyl. Another caveat was that the study was not blind.

Some limitations may be attributed to this study. The first was the lack of a blind study, as the same anesthesiologist was responsible for intraoperative monitoring and postoperative evaluations and knew the treatments employed, because the study was performed in the routine of a private practice, with only one responsible anesthesiologist.

A second limitation is that it is not possible to apply the results obtained in thawed cadavers to fresh ones. According to a consultant anatomist, the spinal cord is one of the first regions to thaw in a cadaver, therefore considering that the cadavers were fully thawed before epidural injections, so was the epidural space. However, the effect of freezing and thawing may change the integrity of epidural anatomical structures, as discussed before.

It is concluded that the dispersion of the solution administered in the lumbar epidural space is smaller in thawed cadavers in relation to alive dogs and, therefore, the results obtained in thawed cadavers cannot be extrapolated to alive dogs in the clinical setting. Lumbar epidural anesthesia provides superior postoperative analgesia and produced longer postoperative sedation to intraoperative fentanyl use in female dogs submitted to ovariohysterectomy.

## Data Availability Statement

All datasets presented for this study are included in the article/Supplementary Material.

## Ethics Statement

The animal study was reviewed and approved by Animal Ethics Committee of São Paulo State University (Unesp), School of Veterinary Medicine and Animal Science. Written informed consent was obtained from the owners for the participation of their animals in this study.

## Author Contributions

DC, LC, FF, and SL performed the experimental design, review, and structuring of the article. DC performed data collection and tabulation. DC and SL performed the statistical analysis. All authors contributed to the article and approved the submitted version.

## Conflict of Interest

The authors declare that the research was conducted in the absence of any commercial or financial relationships that could be construed as a potential conflict of interest.
